# Investigating the Epigenetic Discrimination of Identical Twins Using Buccal Swabs, Saliva, and Cigarette Butts in the Forensic Setting

**DOI:** 10.3390/genes9050252

**Published:** 2018-05-14

**Authors:** Athina Vidaki, Vivian Kalamara, Elena Carnero-Montoro, Timothy D. Spector, Jordana T. Bell, Manfred Kayser

**Affiliations:** 1 Department of Genetic Identification, Erasmus MC University Medical Center Rotterdam, 3015 CN Rotterdam, The Netherlands; a.vidaki@erasmusmc.nl (A.V.); p.kalamara@erasmusmc.nl (V.K.); 2 Department of Twin Research and Genetic Epidemiology, King’s College London, London SE1 7EH, UK; elena.carnero_montoro@kcl.ac.uk (E.C.-M.); tim.spector@kcl.ac.uk (T.D.S.); jordana.bell@kcl.ac.uk (J.T.B.)

**Keywords:** forensics, epigenomics, individual identification, monozygotic twins, DNA methylation, Illumina 450K array, MethyLight, buccal cells, saliva, cigarette butts

## Abstract

Monozygotic (MZ) twins are typically indistinguishable via forensic DNA profiling. Recently, we demonstrated that epigenetic differentiation of MZ twins is feasible; however, proportions of twin differentially methylated CpG sites (tDMSs) identified in reference-type blood DNA were not replicated in trace-type blood DNA. Here we investigated buccal swabs as typical forensic reference material, and saliva and cigarette butts as commonly encountered forensic trace materials. As an analog to a forensic case, we analyzed one MZ twin pair. Epigenome-wide microarray analysis in reference-type buccal DNA revealed 25 candidate tDMSs with >0.5 twin-to-twin differences. MethyLight quantitative PCR (qPCR) of 22 selected tDMSs in trace-type DNA revealed in saliva DNA that six tDMSs (27.3%) had >0.1 twin-to-twin differences, seven (31.8%) had smaller (<0.1) but robustly detected differences, whereas for nine (40.9%) the differences were in the opposite direction relative to the microarray data; for cigarette butt DNA, results were 50%, 22.7%, and 27.3%, respectively. The discrepancies between reference-type and trace-type DNA outcomes can be explained by cell composition differences, method-to-method variation, and other technical reasons including bisulfite conversion inefficiency. Our study highlights the importance of the DNA source and that careful characterization of biological and technical effects is needed before epigenetic MZ twin differentiation is applicable in forensic casework.

## 1. Introduction

Human genetic variation—in particular, the use of short tandem repeat (STR) markers—allows for individual identification of known persons, such as donors of biological traces found at crime scenes [1,2]. The general principle of forensic DNA profiling is the comparison of an STR profile established from trace DNA of an unknown person collected from the crime scene against STR profiles established from reference DNA of known persons, such as convicted crime perpetrators whose STR profiles are stored in the national forensic DNA database. A complete match between an STR profile from a crime scene trace and that of a known person indicates that the matching person is indeed the trace donor. However, except for extremely rare cases [3], such conventional forensic DNA profiling fails to discriminate monozygotic (MZ) twins from within a given pair, which can be forensically relevant in both criminal and paternity casework. This challenge is due to the fact that MZ twins from the same pair derive from the same zygote, hence sharing an ‘identical’ genome sequence and the same forensic STR profile. In forensic cases where there is no other evidence available, courts will have no choice but to continue ruling towards setting prime suspects free [4,5], because no individual conclusion can be derived from DNA evidence. Therefore, a suitable molecular approach needs to be developed to differentiate MZ twins from crime scene stains to be able to solve such criminal cases.

Based on theoretical considerations [6] and recently demonstrated using ultra-deep, whole-genome DNA sequencing (WGS) [7], it was previously predicted that individual-specific, but extremely rare, somatic mutations in form of single-nucleotide polymorphisms (SNPs) can possibly occur at some point during early development in one, but not the other, MZ twin individual; these can be arguably used for MZ twin differentiation. The main drawbacks of this genetic approach to discriminating MZ twins from each other includes the rarity (if they exist at all) of these SNPs, making it very difficult to locate them across the genome; it is only possible when ultra-deep, highly expensive WGS is applied. Moreover, because these SNPs need to occur early in cell development to be MZ twin differentiating, not all cell types used for forensic trace analysis will carry the respective twin-differentiating SNP allele. The risk of investing in ultra-deep WGS and not succeeding in finding any informative twin-differentiating SNPs in the tissue type of the crime scene trace makes the implementation of this genetic approach in routine forensic casework impractical.

In contrast to the stable genome, the epigenome, including various DNA modifications such as DNA methylation, is dynamic and interchangeable in response to various environmental and stochastic events [8,9]. Epigenetic studies using phenotypically concordant and discordant MZ twins have been used in medical research as they account for genetic effects, and are therefore considered ideal for studying environmental influences on health and disease [10,11,12,13,14]. Observed epigenetic drifts within MZ twin pairs can be seen not only at specific genomic loci [15,16], but also at a global level [12], which has unraveled epigenetics’ role in development, ageing, and disease [17,18,19,20,21,22]. The potential of DNA methylation profiling for the discrimination between MZ twins in the forensic setting has already been proposed [23,24,25,26,27,28]; however, these previous studies mainly included reference-type DNA such as whole blood or buccal swabs. Investigations were not extended to trace-type DNA such as from blood or saliva stains, while combining both parts is needed in the forensic setting. Using small sets of MZ twin pairs and applying genome-wide methylation techniques like the Illumina Infinium Human Methylation BeadChip microarrays or methylated DNA immunoprecipitation, forensic researchers identified a number of differentially methylated genomic regions in blood [23,24,25] using diverse methylation difference thresholds. Most of these were located within CpG islands (<500 bp long, >55% GC content, [29]), indicating their potential involvement in gene regulation. Together with blood, twin-to-twin methylation differences have also been investigated in buccal cells, but mainly using more targeted approaches and studying satellite DNA and interspersed repeats. Global LINE-1 methylation analyzed via pyrosequencing was found to be differentially methylated in a small proportion of the studied MZ twin pairs, and significantly correlated with age [26]. In another study, *Alu* methylation analyzed via high-resolution melting curve analysis showed potential for MZ twin differentiation, but the low resolution and the required large sample volume bring its forensic applicability into question [27].

MZ twin studies examining the extent of epigenetic differences, the degree that these are shared between difference pairs, and the feasibility of the epigenetic-based approach in forensic casework are limited thus far. In a recent study, we investigated epigenetic differentiation of MZ twins in a forensic setting in blood by applying a twin pair-specific approach to 10 MZ twin pairs [30]. Using genome-wide microarray-based screening in high-quality and -quantity reference-type blood DNA, we discovered 19–111 twin differentially methylated sites (tDMSs) per MZ twin pair (methylation difference threshold of >0.3). However, only two of the top three tDMSs per pair were successfully validated in the same blood DNA samples by means of methylation-specific SYBR Green-based quantitative (q)PCR. Moreover, two-thirds of the validated tDMSs showed substantial differences in low-quality and -quantity trace-type DNA from small bloodstains. These findings indicate that there are various sources of technical and biological variation, one of them being the DNA source as DNA methylation can be tissue-specific [31,32,33,34] and affected by different cell type compositions [35,36,37].

In the present study, we extended our investigation on the epigenetic differentiation of MZ twins to those biological materials most often confronted within criminal casework, namely buccal swabs, cigarette butts, and saliva. Following the forensic setting, we used buccal swab DNA as reference-type material to resemble high-quality and -quantity reference DNA obtained from buccal swabs collected as reference material in forensic casework. Also, saliva and ‘used’ cigarette butts were collected as trace-type material to resemble low-quality and -quantity trace DNA obtained from this evidence type at crime scenes. Keeping with the forensic setting, we used one MZ twin pair, because in a given forensic case only one MZ pair will be investigated. Moreover, because DNA methylation differences between the genetically identical MZ twins are assumed to be pair-specific due to individualized environmental differences [38], investigating more pairs would not increase the knowledge reliability per tDMSs used, as every MZ pair will have different tDMSs [30]. As an analog to a forensic case, we performed the discovery of candidate tDMSs in reference-type buccal cell DNA via genome-wide DNA methylation microarray analysis; this was followed by candidate tDMS analysis in trace-type DNA from saliva and cigarette butts using the TaqMan-based quantitative PCR (qPCR) method (MethyLight), which, in contrast to microarrays, is sensitive enough for low-quality and -quantity trace DNA analysis. To the best of our knowledge, this is the first study addressing epigenetic MZ twin differentiation in the forensic setting using epithelial materials and applying a combination of genome-wide and targeted DNA methylation analysis.

## 2. Materials and Methods

### 2.1. Sample Collection

This study was approved by the St Thomas’ Hospital Local Ethics Committee (07/H0802/84, 10.09.2010), and participants provided signed informed consent prior to sample collection. The pair of twins used in this study are European female MZ twins, aged 52.5 years. The monozygosity of the twins was confirmed by genotyping 15 highly polymorphic STR loci using the AmpFLSTR™ Identifiler™ PCR Amplification kit (Thermo Fisher Scientific, Waltham, MA, USA). Both twins donated buccal swabs, saliva samples, and ‘used’ cigarette butts. Saliva was collected in Oragene^®^ RNA (RE-100) tubes (DNA Genotek, Kanata, ON, Canada) following standard manufacturer procedures, with the conditions of absence of eating, drinking, or smoking for at least 30 min prior to collection. Four cigarette butts were also collected by using simulated smoking conditions. The participants were asked to bring the unlit cigarette filter to the lips and inhale through the filter at least 10 times with an interval of 1 min between each inhale. All samples were stored at −80 °C until DNA extraction and analysis.

### 2.2. DNA Sample Preparation

Total DNA from buccal swabs, representing reference-type samples, was extracted using the DNeasy Blood & Tissue kit (QIAGEN, Hilden, Germany) according to the manufacturer’s instructions. Saliva DNA was extracted using the QIAamp^®^ DNA Investigator kit (QIAGEN) by following the protocol: Isolation of Total DNA from Small Volumes of Blood or Saliva. As suggested by Oragene^®^ RNA (DNAGenotek), 100 µL of saliva was first incubated for 1 h at 50 °C prior to extraction. The following modifications were applied in order to further maximize and concentrate the DNA yield; 40 µL of ATL buffer was also added to each saliva sample, solutions were incubated at 56 °C for 2 h, and DNA was eluted in 40 µL of elution buffer. DNA from the cigarette butts was extracted using an adjusted salting-out extraction method [39]. Briefly, cigarette butt papers were placed in lysis buffer and incubated at room temperature for one day. Subsequently, proteinase K and 10% sodium dodecyl sulfate (SDS) were added and solutions were incubated at 55 °C for 3 h and at 37 °C overnight. Next, NaCl was added and the solution was centrifuged to collect the supernatant. Later on, isopropanol was added and after centrifuging the supernatant was removed. This step was repeated with ethanol. The remaining pellet containing the DNA was eluted in 40 µL of RNase-free water. DNA samples were quantified using the Quantifiler^®^ Human DNA Quantification kit (Thermo Fisher Scientific), according to the manufacturer’s protocol.

In addition, eight DNA standards of known methylation levels (0–100%) were prepared from low- and high-methylated genomic DNA (EpigenDx, Hopkinton, MA, USA) and were co-analyzed together with the samples. For genome-wide analysis, 750 ng of extracted buccal cell DNA was bisulfite-converted using the EZ DNA Methylation^™^ Kit (ZymoResearch, Irvine, CA, USA). For qPCR analysis, 200 ng of extracted saliva or standard DNA and 75 ng of extracted cigarette butt DNA (due to lower yield from these samples) were bisulfite-converted with a MethylEdge™ Bisulfite Conversion system kit (Promega, Madison, WI, USA). Bisulfite-converted DNA samples were diluted down to 1 ng/µL prior to qPCR. A bisulfite-converted, fully methylated DNA standard (EpiTect Control DNA, QIAGEN) was used as positive control to prepare a dilution series of eight standards with known concentrations (10–0.0781 ng/µL) and was included in each MethyLight assay.

### 2.3. Genome-Wide DNA Methylation Profiling and Data Processing

Genome-wide DNA methylation profiles used in this study were generated using the Illumina Infinium Human Methylation 450K BeadChip array (Illumina, San Diego, CA, USA). Intensity images were captured using GenomeStudio (2010.3) Methylation module (1.8.5) software (Illumina). The Illumina 450K BeadChip assay allows for quantification of DNA methylation levels at 485,512 CpG dinucleotides. For each CpG site, a β value was estimated, ranging from zero, representing completely nonmethylated, to one, representing completely methylated sites. Here, we considered a total of 345,858 probes for our downstream analyses, as a result of stringent quality control procedures, and following the removal of X/Y chromosome probes and CpG sites with identical methylation profiles (0% difference between the twins). To account for technical biases and nonbiological variation including batch effects, we performed two types of normalization of the 450K array results—subset-quantile (SWAN) [40] and functional normalization (FUNNORM) [41], both implemented in Bioconductor package Minfi [42]. In the normalized datasets, to examine the overall similarity of DNA methylation profiles of MZ twins, we computed the correlation across all CpG sites using Pearson’s correlation (r). Based on the average normalized beta value, CpGs were classified as hypomethylated (average β ≤ 0.3, deficiency of methylated alleles), intermediately methylated (average 0.3 < β < 0.7), and hypermethylated (average β ≥ 0.7, overabundance of methylated alleles).

### 2.4. Candidate Twin Differentially Methylated CpG Site Selection

Genome-wide data normalized by both the FUNNORM and SWAN methods were used to select potential tDMSs by following some quality control steps. Firstly, DNA methylation probes that mapped to multiple locations of the reference sequence (with exact sequence match and within two base pair mismatches, N = 30,970) and probes that contain a nonrare polymorphism in the CG site, minor allele frequency (MAF) >0.1 in the European population from 1000 genomes (N = 24,875), were removed. Secondly, markers demonstrating DNA methylation values lower than 0.15 and higher than 0.85 (extreme methylation states) were excluded as they were considered as high risks for technical errors (38.9% and 40.9% in FUNNORM and SWAN datasets, respectively), as also seen in our previous investigation when analyzing candidate tDMSs in whole blood [30]. Following quality control, the absolute methylation difference for each CpG site within the MZ twin pair was calculated in both datasets. Both FUNNORM and SWAN twin-to-twin methylation differences were categorized by applying different thresholds (≥0.30, ≥0.40, and ≥0.50). For qPCR method development, we only considered tDMSs identified by both normalization strategies.

### 2.5. MethyLight

**Figure A1:**
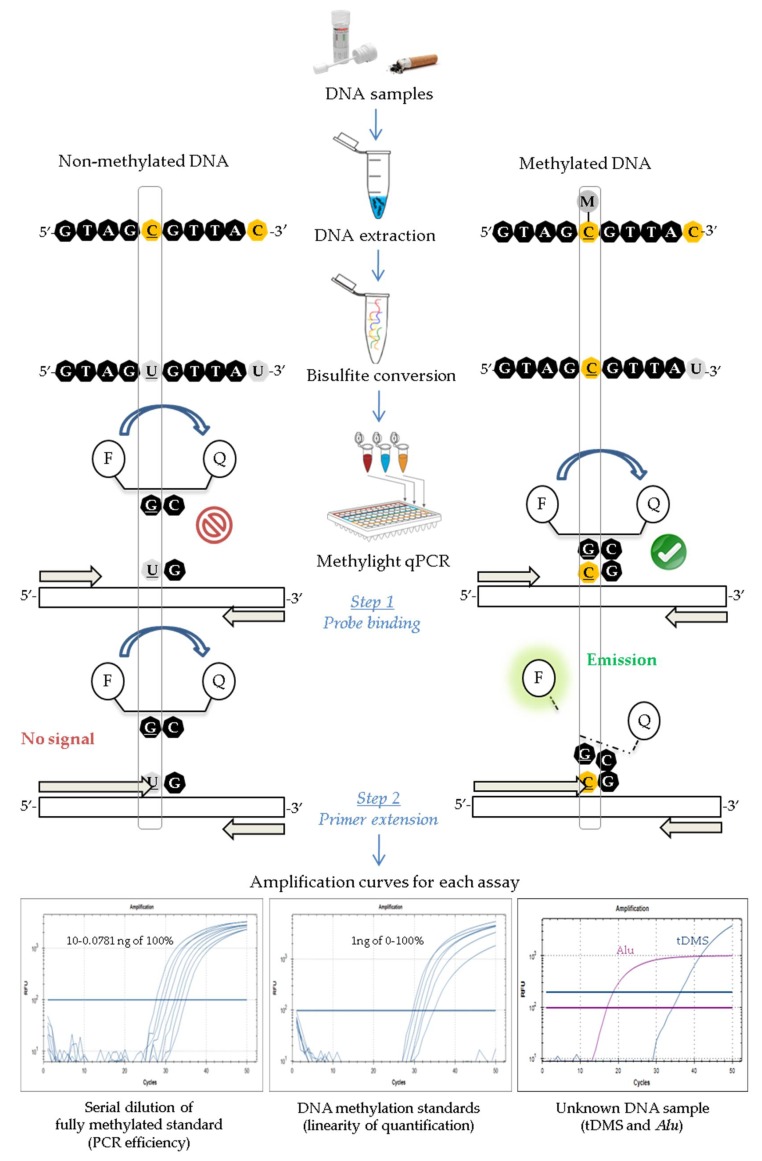
Scheme of targeted DNA methylation analysis using MethyLight.

MethyLight is a TaqMan-based, highly sensitive qPCR technique that relies on the hybridization and cleavage of probes specifically designed to target the CpG of interest [43] (Figure A1). For targeted analysis, the top 25 candidate tDMSs were used for MethyLight assay design. The chromosomal location of the selected tDMSs and the surrounding DNA region were confirmed and extracted using the online Ensemble genome browser (human GRCh37/hg19 genome) [44]. The expected bisulfite-converted DNA sequences were subsequently used to design bisulfite-specific primers for each CpG assay using the BiSearch software [45]. Some of the applied parameters include the primer length (17–26 bp), the PCR product length (<300 bp), and the similarity of primer melting temperature (<5 °C). The selection was based on the assay-specific scores provided by the software and the presence of potential nonspecific PCR product(s). The AutoDimer software [46] was used to assess the formation of primer dimers and hair-pins. Each methylation-specific probe was designed to bind to the sequence that includes the CpG of interest and labelled with the 6-Carboxyfluorescein (6-FAM) fluorophore at the 5′ end. The reference repetitive element *Alu* was used as a methylation-independent control reaction for normalizing the amount of DNA input using previously reported primers [27,31,32]. The *Alu* probe was labelled with Cy5 fluorophore to differ from the CpGs of interest. Due to technical difficulties (CG-rich DNA sequence), no assay could be designed for one tDMS (cg12047941).

The qPCR assays were developed based on the EpiTect MethyLight PCR + ROX^™^ Vial Kit (QIAGEN) and the Bio-Rad CFX96 Touch^™^ Real-Time PCR Detection System (Bio-Rad, Hercules, CA, USA). Each assay was optimized in terms of various parameters including primer/probe annealing temperature and concentration. Two assays (cg11777917 and cg17080283) failed the optimization step due to the generation of nonspecific PCR products, resulting in the successful final development of 23 (22 tDMSs and *Alu*) MethyLight assays (Table A1). Each optimized assay was performed in 10 µL reactions in triplicate, including 5 µL of the 2X EpiTect MethyLight reaction buffer, 1 µL of primer mix (forward and reverse), 1 µL of hybridization methylation-specific probe, 1 µL of 25 µM MgCl_2_, 1 µL of bisulfite converted DNA (corresponding to ~1 ng), and 1 µL of RNase-free water. The final concentration of primer and probes depends on the assay and ranges over 0.4–2 µM and 0.2–1 µM, respectively; this can be found in Table A1. The thermocycling program was set as follows: an initial polymerase activation step at 95 °C for 5 min, followed by 40 cycles of denaturation step at 95 °C for 15 s and annealing/extension step at 55–60 °C for 60 s, depending on the assay (Table A1).

### 2.6. Quantitative PCR Data Analysis

The efficiency (*e*) of each qPCR assay was provided by the standard curve slope of the serially diluted DNA standards (positive controls) according to the equation [47]

(1) e=10−1slope−1.

For each reaction, we obtained the quantification cycle (*C_q_*) value, which describes the cycle required to reach the threshold—set in this case at 100 relative fluorescent units (RFU)—in which fluorescence can be detected. The DNA copy number (*cn*) was calculated using *e* and the average *C_q_*, in the equation

(2) $$cn=e^{-Cq}.$$

Finally, the methylation ratio was calculated using the formula [43,47,48,49]

(3) $$DNA\;methylation\;ratio=\frac{\left(\frac{cn_{CpG}}{cn_{Alu}}\right)sample}{\left(\frac{cn_{CpG}}{cn_{Alu}}\right)fully\;methylated\;standard}.$$

For each sample and standard, the mean and standard deviation of DNA methylation were calculated using the triplicate reactions. Sample reactions with methylation levels falling outside of the range mean ± standard deviation were considered as outliers (technical artifacts due to suboptimal amplification) and, therefore, excluded from analysis. Finally, the detected methylation values of the DNA standards of known methylation levels (0–100%) were used to create the best-fitted linearity curve per assay. The resulting equation based on a minimum of five DNA methylation standards was used to normalize the observed average methylation value of each sample (Table A2).

## 3. Results

### 3.1. Distribution and Twin–Twin Correlation of Genome-Wide Methylation Array Data in Reference-Type Buccal DNA

**Figure 1:**
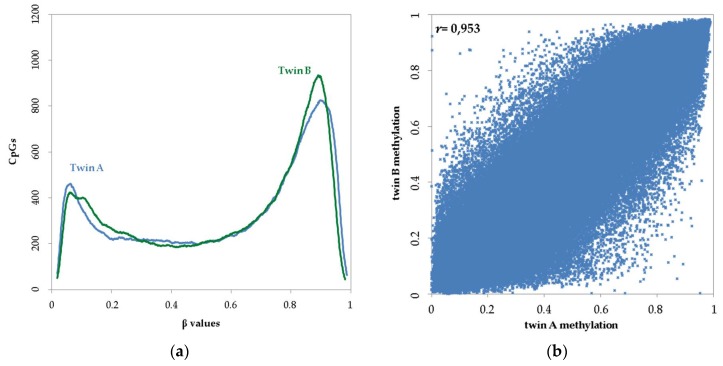
(**a**) Distribution and (**b**) correlation of genome-wide DNA methylation between monozygotic (MZ) twins in buccal cells from Illumina 450K microarrays data using functional normalization.

While the Illumina 450K microarray generally has high reproducibility [50], β values underwent two different normalization approaches to assess technical bias. Considering all CpG sites in buccal cell DNA following quality control and normalization, and by excluding the completely identical ones with 0% twin-to-twin methylation differences, the methylation level demonstrated the typical bimodal distribution for each of the two MZ twin individuals (Figure 1a). Based on our β-value cut-offs taking into account both individuals and normalization strategies, on average, 24.97% of CpGs were classified as hypomethylated (β ≤ 0.3), 26.95% were intermediately methylated (0.3 < β < 0.7), and 48.08% were classified as hypermethylated (β ≥ 0.7). We then explored the correlation between the co-twins considering the CpG sites in the final genome-wide microarray dataset. The observed twin-to-twin correlations (*r*) were 0.953 and 0.962 using functional and subset-quantile normalization, respectively (Figure 1b). The high correlation we observed here in buccal cells from this one MZ pair is slightly lower compared to previous genome-wide estimates from MZ twins in buccal cells (*r* = 0.981–0.994, 10 MZ twin pairs [13]), and also lower compared to those from their whole blood (0.992, [30]). The advanced age of the MZ pair used here at time of sampling (52.5 years) and tissue-specific methylation differences could partly explain these results, as shown previously [11]. The observed high correlation is mainly driven by the vast majority of invariable CpG sites that are either hypo- or hypermethylated in both twins. The small DNA methylation differences between the MZ twins may be attributed in part to stochastic processes and/or MZ discordance for environmental exposures, phenotypes, and diseases, leading to distinct epigenetic changes at the single CpG level [11].

### 3.2. Identification of Candidate Twin Differentially Methylated CpG Sites from Microarray Screening in Reference-Type Buccal DNA

To identify candidate tDMSs in reference-type DNA obtained from buccal swabs, we applied various quality control steps as described in Section 2.4. The number of potential tDMSs resulting from both normalized FUNNORM and SWAN datasets and at different twin-to-twin methylation difference thresholds (≥0.3, ≥0.4, and ≥0.5) is shown in Table 1.

**Figure 2:**
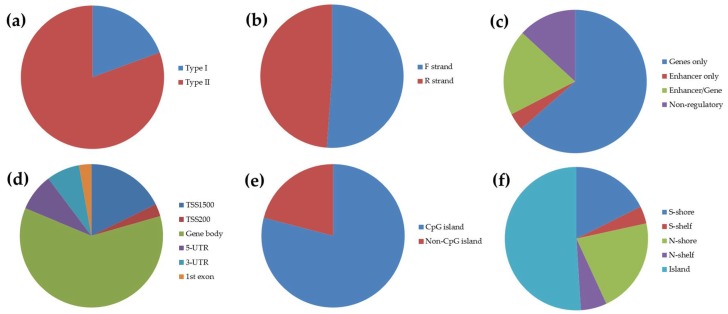
Representation of identified candidate tDMSs with ≥0.4 twin-to-twin methylation difference (*N* = 129) according to (**a**) Illumina probe type, (**b**) DNA strand, (**c**) genomic location, (**d**) location/distribution of gene-associated tDMSs (UTR, untranslated region), (**e**) CpG island, and (**f**) location/CpG density of island-associated tDMSs (N, north; S, south).

As may be expected, the number of candidate tDMSs varied between the two normalization methods, as each accounts for different types of between-sample (FUNNORM) and within-array (SWAN) technical biases [51]. Taking into account CpG sites reaching the threshold values in both datasets, we identified 129 candidate tDMSs showing ≥0.4 twin-to-twin methylation differences (11 of which showing ≥0.5). These findings suggest that tDMSs in buccal cells are limited (0.02% considering all 450K CpGs), yet existing. The majority of candidate tDMSs (83%), found equally on the forward and reverse DNA strands across all 22 chromosomes, were located on or near genes (Figure 2b,c), with more than half located in the gene body (60.1%) and the rest in other function regions like the 5′-untranslated region (5′-UTR) (8.4%) (Figure 2d). Moreover, considering Illumina’s information regarding the 450K probes, 23.3% of the identified tDMSs at this threshold (≥0.4) are associated with enhancer regions, further highlighting their potential involvement in regulating gene expression (Figure 2c). Additionally, the 450K probes have been designed to specifically target CpG islands (CGI) (<500 bp, GC content > 55%, [29]), CGI shores (up to 2 kb from CGI), CGI shelves (between 2 and 4 kb from CGI), as well as non-CpG island regions (>4 kb from CGI). Out of the 129 candidate tDMSs, the vast majority (79.1%) are associated with a CGI (Figure 2e,f). At this cut-off (≥0.4), CGI probes are largely represented, which was also seen in blood [30] and in other tissues [25,28].

### 3.3. MethyLight Method Development for Top Candidate Twin Differentially Methylated CpG Sites and Performance Assessment

**Figure A2:**
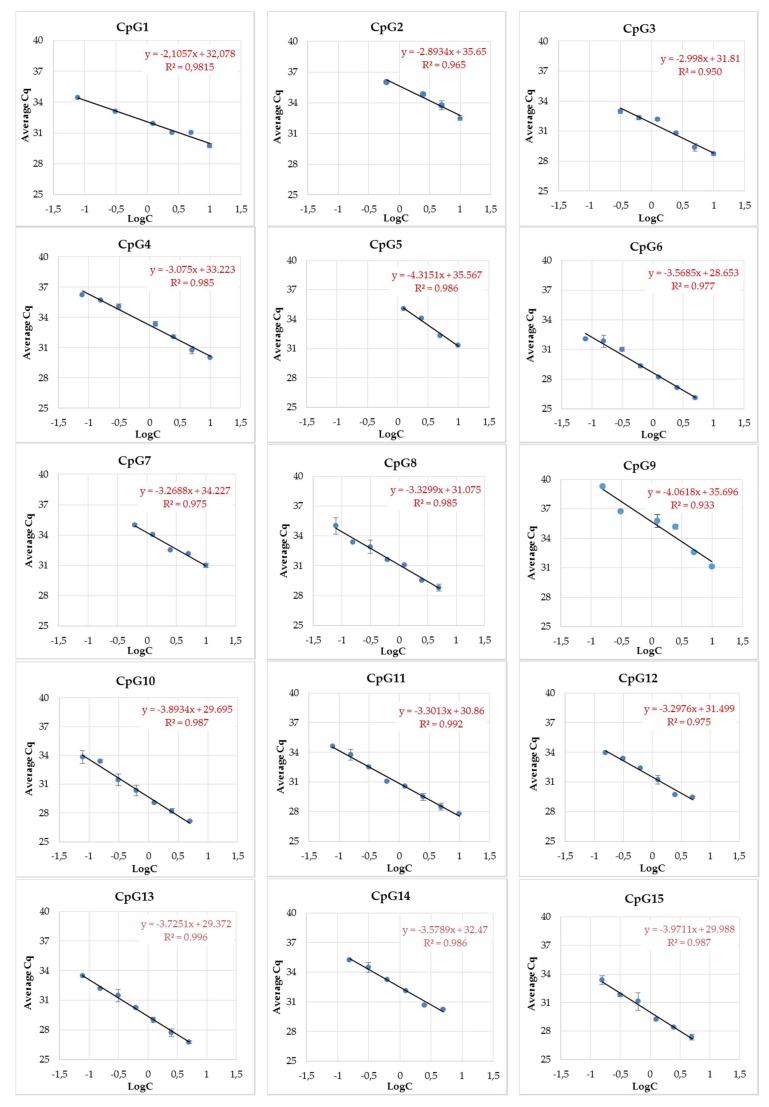

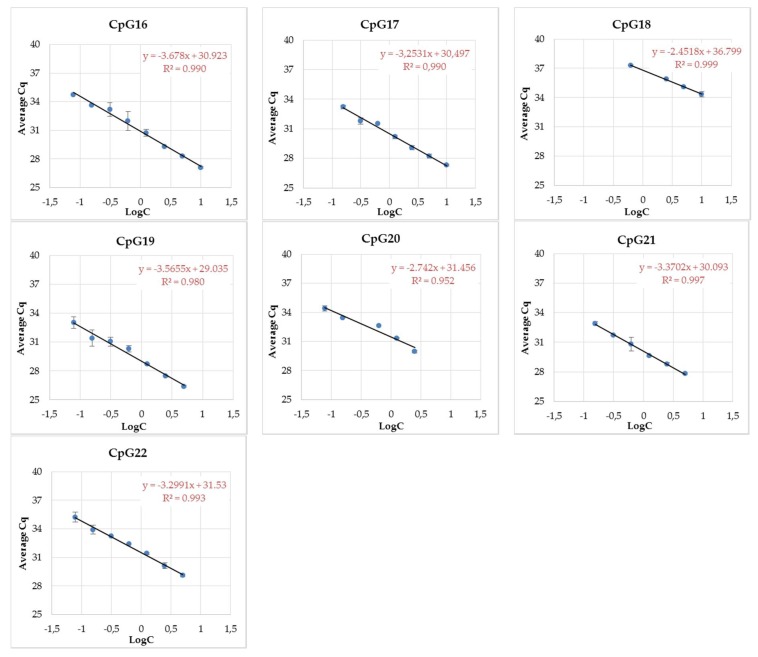
Standard curves of methylated DNA copy number for all MethyLight assays corresponding to the top 22 tDMSs, as generated by using eight standards from a serial dilution of the fully methylated DNA standard (10–0.0781 ng/µL). Each graph plots the average *Cq* against the logarithm of the known DNA concentration, with error bars corresponding to each standard’s standard deviation from the three replicates. The best-fitted linear equation is shown in red.

For qPCR method development, we selected the top 25 candidate tDMSs demonstrating ≥0.5 average twin-to-twin methylation differences in both normalized buccal datasets (Table 2). The sensitivity and efficiency of each MethyLight assay was tested by analyzing eight DNA standards derived from a serial dilution of the fully methylated DNA, with concentrations ranging from 10 ng to 0.078 ng (Table A2). The minimum detected bisulfite-converted, methylated DNA amount for each assay ranged between 0.078 ng and 1.25 ng. More specifically, 50% of the assays resulted in successful amplification when using as little as 0.078 ng of methylated DNA input, whereas 5 out of the 22 assays worked with 0.156 ng of DNA. These results give a strong indication that the selected method for methylation analysis is very sensitive, and comparable with other forensic DNA-based tests. Figure A2 includes the standard curves when plotting the average *C_q_* values against the logarithm of the known DNA concentration, which were linear (R^2^ = 0.933 − 0.999). Moreover, all assays resulted in a high efficiency of bisulfite PCR amplification ranging between 70% and 100%. Particularly, 55% of the assays gave 100% efficiency, whereas 32% resulted in efficiencies higher than 80%.

**Figure A3:**
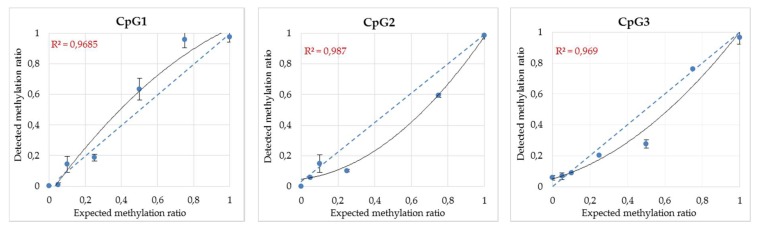

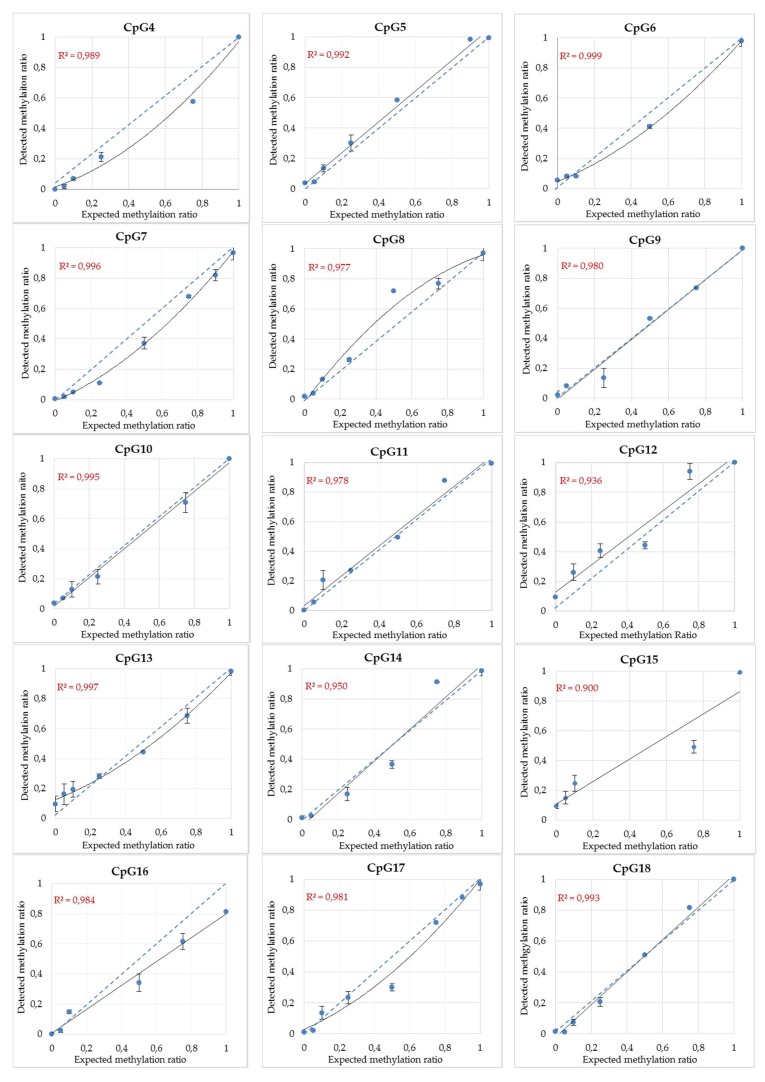

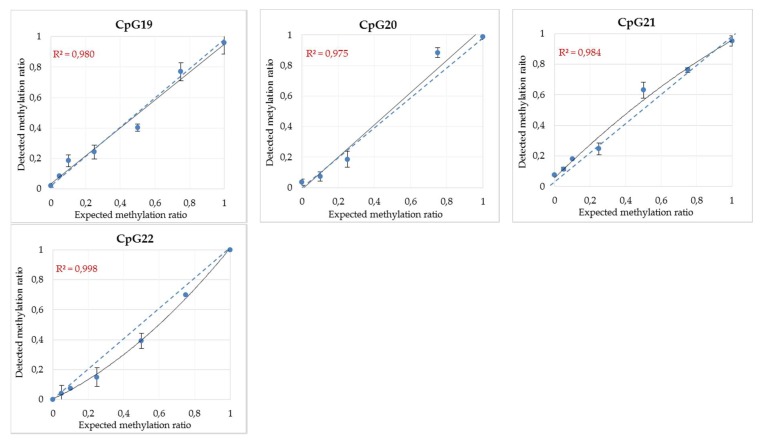
Linearity curves of methylation quantification for all MethyLight assays corresponding to the top 22 tDMSs, as generated by using seven standards with known DNA methylation levels (0–100%). Each graph plots the average normalized methylation ratio against the expected one, with error bars corresponding to each standard’s SD from the three replicates. The R^2^ values of the best-fitted lines (linear or quadratic polynomial) (black lines) are shown in red, while the equations used for normalization are shown in Table A2. Blue dotted lines represent the completely linear line (R^2^ = 1).

We also tested the linearity of methylation quantification, where the detected methylation of commercially available DNA standards with known methylation levels was plotted against the expected methylation values for every assay (Figure A3). In line with the literature [30,52,53], the relationship between observed and expected methylation detection differed across the assays from linear to quadratic polynomial, as a result of different amplification efficiencies and bias. Curves were skewed either above or below the expected linear curve (blue color, Figure A3), indicating preferential amplification of the methylated or nonmethylated allele, respectively. Some of these discrepancies for both the low-methylated (assumed as 0%) and high-methylated (assumed as 100%) DNA standards can also be explained by variable methylation levels or a small degree of incomplete bisulfite conversion. To minimize these effects, the equation derived from each linearity curve was used to normalize the observed methylation values (Table A2). Finally, to test the reproducibility, all qPCR reactions were performed in triplicate. We calculated the average standard deviation, ranging between 0.010 and 0.044 (Table A2). Compared to other methods like SYBR Green-based qPCR [30], pyrosequencing [54], and massively parallel sequencing [52], these values are considered low for methylation assays and are in concordance with other MethyLight studies [55].

### 3.4. Saliva DNA Analysis of 22 Top Twin Differentially Methylated CpG Sites Selected from Microarray Data in Reference-Type Buccal DNA

**Figure 3:**
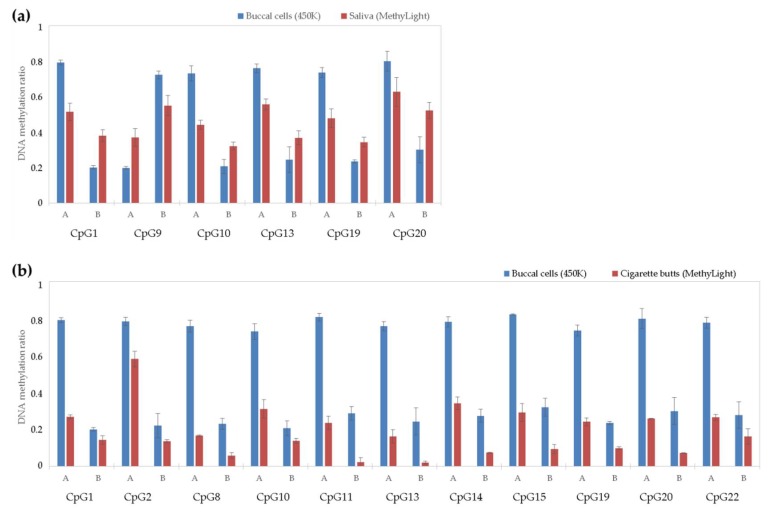
DNA methylation values of top tDMSs selected from microarray data in reference-type buccal DNA (blue) and successfully validated (≥0.1 methylation differences with the same direction) with MethyLight qPCR in trace-type DNA from (**a**) saliva (*N* = 6), and (**b**) cigarette butt DNA (N = 11), (red) for twin individuals A and B. The bars correspond to average detected methylation (triplicate analysis), while error bars indicate observed standard deviation.

**Figure A4:**
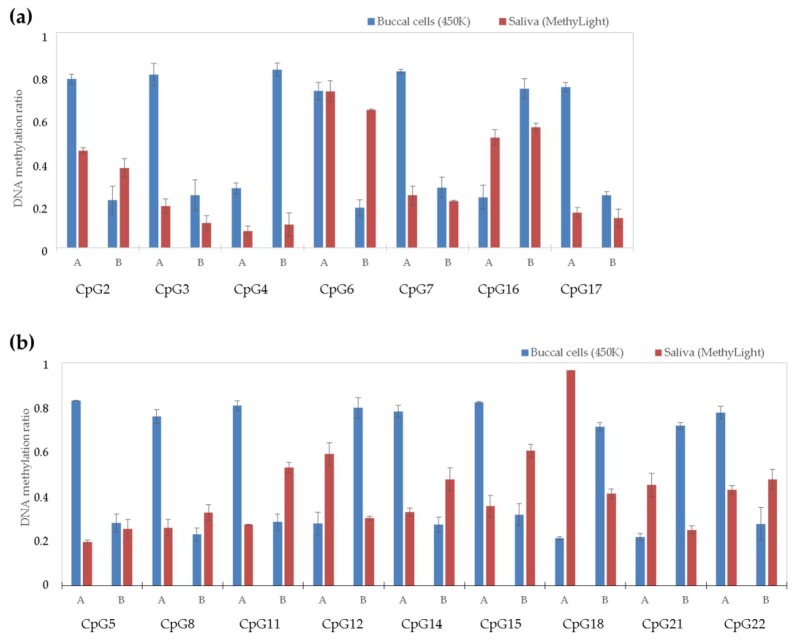

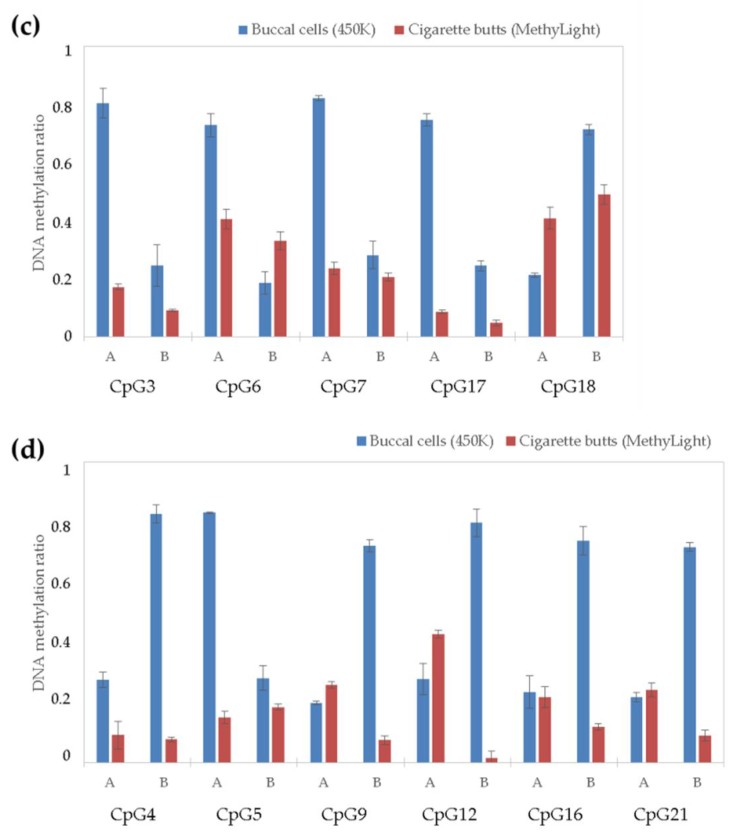
DNA methylation values of top tDMSs selected from microarray data in buccal DNA (blue) and analyzed with MethyLight qPCR in (**a**,**b**) saliva and (**c**,**d**) cigarette butts for twin individuals A and B; (**a**,**b**) tDMSs with <0.1 methylation differences in saliva and cigarette butts but with the same direction as in buccal cells (*N* = 7 and *N* = 5, respectively), (**c**) tDMSs with opposite direction of methylation differences between sample types (false positives) (*N* = 9 for saliva and *N* = 6 for cigarette butts). The bars correspond to average detected methylation (triplicate analysis), while error bars indicate observed standard deviation.

While all of the selected 22 top tDMSs resulted in twin-to-twin absolute methylation differences of ≥0.4 in buccal DNA, none of them reached this cut-off value in saliva DNA using MethyLight. Looking at the raw (average) methylation values per individual and per tDMS, these differ significantly with an average absolute deviation of 0.3 (ranging 0.002–0.756) (Table 3). Only six of the 22 tDMSs (27.3%) resulted in methylation differences greater than 0.1 (average of 0.144) (Figure 3a), with another seven of the 22 tDMSs (31.8%) showing smaller but robust differences (<0.1, average of 0.06) in saliva DNA with MethyLight (Figure A4a,b). For all these 13 tDMSs, the direction of methylation difference was the same as that seen in buccal DNA with microarrays, meaning that the same twin individual showed higher methylation in both buccal cell and saliva DNA. The remaining nine of the 22 tDMSs (40.9%) unexpectedly demonstrated a different direction of twin-to-twin methylation differences (Figure A4c,d). Interestingly, we observed a pattern concerning twin individuals showing low methylation levels in buccal cells, with the latter being much increased in saliva (Table 3).

### 3.5. Cigarette Butt DNA Analysis of 22 Top tDMSs Selected from Microarray Data in Reference-Type Buccal DNA 

As shown in Table 3, the methylation results obtained in cigarette butt DNA are much lower compared to those of both buccal DNA and saliva DNA. As seen with saliva DNA in the same table, while all 22 tDMSs resulted in twin-to-twin absolute methylation differences of ≥0.4 in buccal DNA using the 450K array, none of them reached this cut-off value using MethyLight in cigarette butt DNA. Looking at the raw (average) methylation values per individual and per tDMS, these differ significantly with an average absolute deviation of 0.34 (ranging 0.016–0.783) compared to buccal cell data, and 0.21 (ranging 0.009–0.559) compared to saliva data (Table 3). As shown in Figure 3b, 11 of the 22 tDMSs (50%) resulted in methylation differences greater than 0.1, with 5 of the 22 tDMSs (22.7%) showing smaller, subtle differences (<0.1), and 6 of the 22 tDMSs (27.3%) showing the opposite expected methylation profiles and differences (Figure A4c,d).

## 4. Discussion

The inability to discriminate between identical twins using conventional forensic STR profiling and the challenges in applying ultra-deep whole-genome sequencing to find rare somatic SNPs [7] have led forensic researchers to look for alternative ways to differentiate MZ twins via molecular analysis, with particular attention to DNA methylation profiling [23,25,26]. While DNA methylation occurs in the 5′ carbon of cytosine in 5′-CpG-3′ positions without affecting the DNA sequence itself, such epigenetic differences can be detected with both genome-wide screening and targeted methods. Based on observations derived from genome-wide data, potential candidate markers capable of discriminating identical twins have been reported in blood [24,25] and buccal cells [12]; however, studies on testing the forensic feasibility and applicability of this are lacking. 

In this study, we aimed to expand our previous investigation in whole blood [30] by analyzing other forensically relevant tissues, namely, buccal cells, representing the most typical human biological material collected as reference material in forensic casework, as well as saliva and cigarette butts representing materials often found at crime scenes. We identified tDMSs in buccal DNA in one example MZ pair using the Illumina HumanMethylation450 platform, and analyzed them in saliva and cigarette butt DNA using the targeted MethyLight approach. This setup was chosen to mimic the typical forensic casework setting with one specific MZ twin pair in question. The approach includes the discovery of candidate tDMSs using genome-wide screening technology in reference-type DNA of sufficiently high quantity and quality (here 750 ng) from buccal swabs, and the subsequent analysis using trace-sensitive and targeted technology of the most promising identified tDMSs in trace-type DNA of typically low quality and quantity (here ~1 ng per reaction) from saliva stains and cigarette butts. Due to the high DNA input requirement of the Illumina HumanMethylation450 platform, we were unable to type the trace-type DNA with this technology, as this would generate stochastic loss of probe signals and statistically unreliable data when using suboptimal DNA amounts (<300 ng).

For identifying candidate tDMSs from reference-type buccal cells, the genome-wide DNA methylation microarray data first underwent various quality control steps aiming to minimize the chances of technical errors that could be particularly evident when small methylation differences are expected, as in the case of MZ twins. To this end, we applied different cutoffs of absolute methylation difference, which resulted in a pool of 129 candidate tDMSs showing differences larger than 0.4. During our analysis, we avoided not only problematic CpG sites due to nonspecific probes or probes containing common SNPs, but also CpG sites demonstrating an *on/off* methylation pattern when comparing the twins. As shown in Figure 1a, the differences between the two MZ twin individuals are quite large for CpGs showing very low (<0.15) and very high (>0.85) methylation values; hence, these were excluded from the top candidate choice as they could contain potential technical errors as a result of failed or suboptimal performance of one of the two Illumina probes used in the microarray technology. Justification of this approach is provided by our previous study using blood [29], where the proportion of candidate tDMSs not successfully validated was considerably higher when including candidate tDMSs with large twin-to-twin methylation differences in the microarray data, relative to the validation rate when excluding those, as also applied here. Although we cannot completely explain this, excluding such CpGs from further analysis avoids one possible error source. Future empirical data are necessary to further document these potential technical biases related to extreme methylation states.

The second step in our study was to develop a targeted analysis method for the identified candidate tDMSs markers that provides robust, reproducible, and sensitive DNA methylation detection suitable for low-quality and low-quantity trace-type DNA. While in our previous investigation of MZ twin differentiation from blood [29] we successfully applied a SYBR Green-based qPCR method, in the present study we aimed to improve the accuracy, specificity, and sensitivity of the trace DNA analysis method by taking advantage of the TaqMan technology. MethyLight has been successfully used for both singleplex and small-scale methylation analysis before [48,49,55], and was applied for the purpose of this investigation. An initial method assessment gave very promising results, including <1 ng minimum bisulfite-converted DNA input (which is considered the optimal DNA input into PCR in various commercially available STR kits) and <5% standard deviation of the detected methylation. Each qPCR MethyLight assay has a PCR efficiency ranging from 70% to 100%, which depends on (i) the DNA sequence itself (CG-rich sequences might be more difficult to amplify); (ii) the specificity of the genomic sequence; (iii) the length of the PCR product (as bisulfite-converted DNA is often fragmented); and other technical reasons. Nevertheless, we accounted for the PCR efficiency in our DNA methylation ratio calculations as explained in Section 2.6. However, due to the singleplex nature of the method, there are associated limitations in terms of total required DNA input and time required for method development. 

The third step in our study was to verify whether the same methylation differences as seen in the twins’ buccal cells with microarrays can also be observed in DNA from saliva and ‘used’ cigarette butts with trace-sensitive MethyLight qPCR. Generally, we observed much lower methylation levels in both saliva and cigarette butt DNA with MethyLight, which, as a consequence, resulted in much lower twin-to-twin differences than observed in buccal DNA with microarrays. We also observed the opposite twin-to-twin methylation differences for a large proportion of our candidate tDMSs, potentially introducing false positives in a forensic investigation (identifying the wrong twin individual). Our inability to verify the buccal-derived methylation data can be explained by various technical factors in either platform. Despite our efforts to correct them via normalization strategies (also seen in blood [29]), errors based on method-to-method discrepancies between genome-wide and targeted methods, difference amplification chemistries and bias, Illumina probe-specific suboptimal performance, and incomplete bisulfite conversion can lead to alternative results. In the case of cigarette DNA, together with the factors mentioned above, we consider that lowering the DNA input for bisulfite conversion used for these samples due to non-availability (75 ng) could have an impact on our analysis, as it falls outside of the manufacturer’s optimal DNA input range. Nevertheless, from a forensic perspective, this amount is considered sufficient.

The observed discrepancies, however, can also be explained by biological variation, as introduced by differential cell-type composition in each of these sample types. While for genetic analysis cell composition is often not relevant, for epigenetic analysis the tissue of origin is of crucial importance. It is known that DNA methylation is altered between tissues, not only at a genome-wide level but also at the single CpG level; therefore, samples with different cell type compositions can demonstrate different DNA methylation profiles [56,57]. At the single CpG level, DNA methylation is a binary variable, meaning that at any given CpG site each cell might either be methylated or nonmethylated. However, the observed DNA methylation ratio as obtained from lab techniques is a result of the combined methylation profiles of all cells included in the sample. While the vast majority of cells contained in saliva are of epithelial origin, studies have shown that the cellular content includes erythrocytes, leukocytes, epithelial cells, and bacteria [13,57]. Similarly, considering the normal use of cigarettes during smoking, one expects that since cigarette butts come in contact with the mouth, there is a high probability that obtained DNA originates from saliva (buccal cells and leukocytes) together with epithelial skin cells from the lips. This study demonstrated that this ascertainment is crucial and has to be taken into account in future forensic scenarios, especially when matching reference-trace samples are not available. Given that pure buccal-cell-based forensic material are not likely to be present at crime scenes, while buccal swabs represent the most commonly used reference material in forensic casework, such tissue effects have to be carefully documented. Ideally, and to reduce or avoid epigenetic cell type effects, reference sample collecting strategies should be adaptable towards the trace sample; for example, collecting and using saliva reference samples in cases where saliva trace samples are found at the crime scene.

One limitation of our study was that the DNA samples used for reference-type DNA microarray analysis were no longer available for qPCR testing of the microarray-derived candidate tDMSs. Unfortunately, in our study, such a technical validation step was impossible due to the exhaustion of buccal DNA during the microarray analysis (750 ng of extracted DNA due to the method’s requirements; only one buccal swab was collected). In a forensic setup, this is avoided as typically several buccal swabs are collected as reference material; this increases the total amount of reference DNA available and thus allows such technical validation. Initially, we reasoned that the lack of buccal DNA for qPCR testing could be compensated by using saliva DNA. Based on prior knowledge at the time of sample collection, saliva DNA methylation was not expected to be so different from buccal cell DNA methylation. Such sample type DNA methylation differences have only been highlighted in a more recent study [54] as well as in our study here. In our previous study on whole blood, we did have DNA used for microarray analysis also available for qPCR validation testing simply because the previously collected blood sample provided much more DNA than the buccal swab collected here. There we found that about one-third of the microarray-derived candidate tDMSs strongly deviated in their qPCR results relative to the microarray data when tested in the very same DNA samples, which is explained by method-to-method discrepancies [29]. However, in that study, we also demonstrated that from those tDMSs that were successfully technically validated, about one-third strongly deviated in their blood trace DNA results relative to the reference blood DNA data using the same qPCR method. This previous finding suggests that a proportion of the discrepancies we observed in the present study between buccal array data and saliva/cigarette butts may be caused by method issues, but very likely not all of those we observed. Moreover, the discrepancies we found here between saliva and cigarette butts cannot be explained by method issues as for both forensically relevant sample types we used the same qPCR approach. In order to make stronger conclusions about systematic patterns of tissue-specific DNA methylation in our investigated CpGs, more individuals or twin pairs need to be investigated. However, even in that case, DNA methylation differences between the MZ twins are expected to be pair-specific due to individualized environmental differences; therefore, the candidate tDMSs will very likely always be different in every forensic scenario.

From the statistical perspective, it is currently unknown what the minimum number of tDMSs to be analyzed is to reach a statistically robust identification of twin individuals, but it is likely to be specific to tissue, twin pair, and forensic case circumstances. If more markers are to be investigated, other methods allowing for large-scale multiplexing, such as massively parallel sequencing, may be more suitable. In general, a technology switch from reference-type DNA methylation screening analysis to trace-type targeted DNA methylation analysis is necessary because the DNA methylation microarray technology used in high-quality and high-quantity reference-type DNA such as from buccal cells (or whole blood [29]) is unlikely to work in low-quality and low-quantity trace-type DNA such as from saliva and cigarette butts (or bloodstains [29]). In the future, given that there is no current method for accurately quantifying bisulfite-converted DNA and that the experimental design relies on estimates regarding bisulfite DNA recovery provided by the manufacturer, introducing such a quantification step after bisulfite conversion will likely improve the accuracy and reproducibility of the targeted DNA methylation analysis. Moreover, recently there have been a few studies published proposing a cell type composition scoring in complex tissues as a quality step prior to DNA methylation analysis [37,58], which can also be adopted in this case to explain DNA methylation differences by cell type differences as assumed based on the results presented here. For example, this approach could work by analyzing a small number of certain CpGs with known expected methylation levels, for calculating the proportion of leukocytes in saliva and correct saliva-based methylation values prior to comparison with buccal-cell-derived data, as recently demonstrated [37,58]. Lastly, to account for tissue-specific methylation effects more effectively, a potential strategy could be to use tissue-shared tDMSs, which, however, remain to be identified in future MZ twin studies involving various forensically relevant human materials. 

## Figures and Tables

**Table 1:** Potential twin differentially methylated CpG sites (tDMSs) derived from genome-wide microarray screening following two normalization strategies (functional—FUNNORM and subset-quantile—SWAN) and three methylation difference thresholds.

Normalization Method	Number of Potential tDMSs (% of Total Analyzed CpGs)		
Threshold	> 0.495	>0.395	>0.295
FUNNORM	239 (0.07%)	998 (0.29%)	4.208 (1.22%)
SWAN	66 (0.02%)	411 (0.18%)	2.068 (0.60%)
Shared by both methods	61 (0.02%)	363 (0.11%)	1.751 (0.51%)

**Table 2:** Top 25 candidate tDMSs selected for MethyLight quantitative (q)PCR assay design.

CpG	Assay	FUNNORM			SWAN			Average Diff
		A	B	Diff	A	B	Diff	
**cg01115923**	CpG1	0.801	0.207	0.594	0.783	0.193	0.590	**0.592**
**cg23449764**	CpG2	0.768	0.173	0.595	0.799	0.266	0.533	**0.564**
**cg14525379**	CpG3	0.769	0.194	0.575	0.841	0.295	0.546	**0.561**
**cg26857315**	CpG4	0.258	0.850	0.592	0.294	0.807	0.513	**0.553**
**cg18812079**	CpG5	0.831	0.310	0.521	0.833	0.252	0.581	**0.551**
**cg07033292**	CpG6	0.757	0.157	0.600	0.700	0.212	0.488	**0.544**
**cg17434062**	CpG7	0.815	0.247	0.568	0.827	0.314	0.513	**0.541**
**cg11777917**	(-) **^1^**	0.841	0.245	0.596	0.749	0.279	0.470	**0.533**
**cg17854471**	CpG8	0.782	0.209	0.573	0.737	0.25	0.487	**0.530**
**cg13038544**	CpG9	0.193	0.737	0.544	0.202	0.707	0.505	**0.525**
**cg23041250**	CpG10	0.760	0.178	0.582	0.699	0.234	0.465	**0.534**
**cg12047941**	(-) **^1^**	0.800	0.210	0.590	0.743	0.289	0.454	**0.522**
**cg14737704**	CpG11	0.823	0.312	0.511	0.793	0.26	0.533	**0.522**
**cg18562578**	CpG12	0.242	0.831	0.589	0.314	0.766	0.452	**0.521**
**cg05415840**	CpG13	0.742	0.192	0.550	0.777	0.294	0.483	**0.517**
**cg17080283**	(-) **^1^**	0.719	0.157	0.562	0.691	0.224	0.467	**0.515**
**cg20482280**	CpG14	0.802	0.249	0.553	0.764	0.298	0.466	**0.510**
**cg15904939**	CpG15	0.826	0.353	0.473	0.821	0.285	0.536	**0.505**
**cg03353765**	CpG16	0.196	0.706	0.51	0.274	0.772	0.498	**0.504**
**cg03571301**	CpG17	0.762	0.256	0.506	0.731	0.232	0.499	**0.503**
**cg02961798**	CpG18	0.206	0.701	0.495	0.217	0.726	0.509	**0.502**
**cg00134667**	CpG19	0.756	0.228	0.528	0.715	0.240	0.475	**0.502**
**cg02886509**	CpG20	0.838	0.247	0.591	0.761	0.351	0.410	**0.501**
**cg13460168**	CpG21	0.207	0.729	0.522	0.229	0.708	0.479	**0.501**
**cg10399269**	CpG22	0.789	0.226	0.572	0.757	0.329	0.428	**0.500**

^1^ Excluded from analysis due to unsuccessful method development.

**Table 3:** Methylation levels for 22 top tDMSs selected from microarray data in buccal DNA and analyzed via MethyLight qPCR in saliva and cigarette butt DNA.

Twin	Top CpG	Methylation Ratio			Top CpG	Methylation Ratio		
		Buccal ^1^	Saliva ^2^	Cigarettes ^2^		Buccal ^1^	Saliva ^2^	Cigarettes ^2^
**A**	**1**	0.792	0.513	0.268	**12**	0.278	0.591	0.428
**B**		0.200	0.378	0.144		0.799	0.303	0.016
**A**	**2**	0.784	0.452	0.581	**13**	0.760	0.557	0.162
**B**		0.220	0.371	0.135		0.243	0.367	0.020
**A**	**3**	0.805	0.193	0.171	**14**	0.783	0.330	0.341
**B**		0.245	0.115	0.091		0.274	0.477	0.074
**A**	**4**	0.276	0.078	0.092	**15**	0.824	0.357	0.292
**B**		0.829	0.107	0.077		0.319	0.605	0.093
**A**	**5**	0.832	0.195	0.151	**16**	0.235	0.513	0.219
**B**		0.281	0.254	0.185		0.739	0.559	0.119
**A**	**6**	0.729	0.727	0.405	**17**	0.747	0.164	0.086
**B**		0.185	0.640	0.331		0.244	0.137	0.047
**A**	**7**	0.821	0.244	0.234	**18**	0.212	0.968	0.408
**B**		0.281	0.216	0.205		0.714	0.413	0.490
**A**	**8**	0.760	0.260	0.165	**19**	0.736	0.477	0.241
**B**		0.230	0.327	0.057		0.234	0.343	0.099
**A**	**9**	0.198	0.368	0.259	**20**	0.800	0.627	0.259
**B**		0.722	0.549	0.075		0.299	0.523	0.072
**A**	**10**	0.730	0.440	0.311	**21**	0.218	0.452	0.243
**B**		0.206	0.320	0.138		0.719	0.249	0.090
**A**	**11**	0.808	0.274	0.235	**22**	0.778	0.429	0.265
**B**		0.286	0.530	0.023		0.278	0.477	0.162

^1^ Buccal methylation values were derived from normalized Illumina 450K microarray dataset. ^2^ Saliva and cigarette methylation values were derived from MethyLight qPCR.

## Appendix A

**Table A1:** MethyLight TaqMan-based qPCR assays for the top 22 tDMSs selected from DNA methylation microarray data in buccal DNA for one MZ twin pair.

Assay	CpG	Primer Sequence (5′-3′) ^1^		Primer Length (bp)	Ta (°C)	Primer Concentration (µM)	PCR Length (bp)
CpG1	cg01115923	F	TTTTTTTGTTTATAGTGGGAG	21	60	2/1	86
		R	ACTACTAATCCCAAAACTAAAAA	23			
		Pm	CCRTACCRAAAACTATAAC**G ^2^**	20			
CpG2	cg23449764	F	TGTGTGATAGTGAGAAGTATAAA	23	60	2/1	250
		R	AACTTCTAAATCCAATACCA	20			
		Pm	CGAAATAAACATATATAATAACAC**G**	25			
CpG3	cg14525379	F	GGGTTTATAGATTGTTTTTAGGT	23	60	2/1	249
		R	AAATCTCCTTTACCCTTTTACTT	23			
		Pm	AACAAATATAATAAACTTAAATACC**G**	26			
CpG4	cg26857315	F	TTTAGGAGGGAAGTATAGGAA	21	58	1.2/0.6	286
		R	CCCAAAATACTAAAAAACCAAA	22			
		Pm	AAATAAATAAACGCGTCCC**G**	20			
CpG5	cg18812079	F	GTATTTAGAGGAGTAGATA	19	58	1.2/0.6	143
		R	TACACCTAAAAAAAATCCCA	20			
		Pm	CTAAAAACCCAATCCTACC**G**	20			
CpG6	cg07033292	F	TGTTGATAGTTGTATAGTAG	20	57	0.8/0.4	120
		R	ATAACTAAAAAACCCAACC	19			
		Pm	ACCRTAATTAAAACCAAAACC**G**	22			
CpG7	cg17434062	F	TTTTTTGAGGTAGTGTA	17	59	1.2/0.6	167
		R	RCTTCCCCAAAATAAAAATAATC	23			
		Pm	AAATTCCAATATCTAAAATACCC**G**	24			
CpG8	cg17854471	F	GTTGTGTTAGTTATTTATTTTTGGG	25	57	0.8/0.4	224
		R	TAAAACCAACCTCATTCTT	19			
		Pm	AAAAATTAACACTATACTATCAAC**G**	25			
CpG9	cg13038544	F	GGGGGAATTAGGTATTATTTTTA	23	57	1.2/0.6	248
		R	CAAATATAAAAACCCTACTC	20			
		Pm	CGTAACAAAATAAAATCCGCTC**G**	23			
CpG10	cg23041250	F	GGTTTTTTTTTTAGGTGT	18	57	0.8/0.4	177
		R	AAAACCTCACCCTAACCTAA	20			
		Pm	ACCTCRCCCTCCACRC**G**	17			
CpG11	cg14737704	F	AATTGTTGTGTGTTGGTGGATA	22	55	0.8/0.4	220
		R	ACCAACAATAATAAAAAC	18			
		Pm	AATAAAACTAATAAACTCCAC**G**	22			
CpG12	cg18562578	F	GAGGATTTTTGTTTGGTTTTT	21	59	0.8/0.4	255
		R	RTAACTCCCTTTCTATATAT	20			
		Pm	CTTCTTTACCAACCACRATAC**G**	22			
CpG13	cg05415840	F	ATTTTTGAGTTGGGGTTGATT	21	55	0.8/0.4	142
		R	CCCTACAAAAAAAAAAACT	19			
		Pm	ACATCTAAATAACACRAATATATAC**G**	26			
CpG14	cg20482280	F	TTGGTTGTTTAGGAAGTGTAT	21	56	0.8/0.4	262
		R	AATCTTCCTATAAACAAAAA	20			
		Pm	TCTCCTCTATAACCATATAAAACAC**G**	26			
CpG15	cg15904939	F	TTTTTAAGGTTGTGAGTTAG	20	56	0.8/0.4	142
		R	ACTAACCCTACTAAAATA	18			
		Pm	AATAACCTAAAATTATCCACCAC**G**	24			
CpG16	cg03353765	F	TTAGTGGATAGGAAAGTTAA	20	60	1.2/0.6	216
		R	TTTCAAACAACACAAAAACC	20			
		Pm	CGACTACCAAATAAAAACTACTTAC**G**	26			
CpG17	cg03571301	F	ATTTGTGAATAGTATTATGGGGA	23	60	1.2/0.6	231
		R	CATTTCTCTACCAACAAAAA	20			
		Pm	CCATCCCTATATTTACTAAC**G**	21			
CpG18	cg02961798	F	GGTTGTTYGGTATTTTTTAGTAGT	24	58	1.2/0.6	191
		R	ATCCTAACTTCTTCCTA	17			
		Pm	CRAAATATACCTAAATATAAAACTCC**G**	27			
CpG19	cg00134667	F	AGGAGGGATTTTTTTTAAGGTA	22	56	0.8/0.4	146
		R	AAAAATACCCAACTCTATCT	20			
		Pm	AAATTAATACTTTCCAAATACC**G**	23			
CpG20	cg02886509	F	GGAATATTTGTGGGTAAATT	20	58	0.8/0.4	222
		R	RCCACTACTACTTTATTCTCTAA	23			
		Pm	CTCAAAAATCATCACRTCC**G**	20			
CpG21	cg13460168	F	TTTTTGATATTTTTGTGGGTGG	22	60	0.8/0.4	187
		R	ATCCCCCRAATTTTATTCTTAAC	23			
		Pm	TCCAAACTTAACAATAAATAAC**G**	23			
CpG22	cg10399269	F	TTTTTTTATGGTTTGTTGGT	20	57	0.8/0.4	196
		R	AACCTCTATAACCTCAAAAT	20			
		Pm	CRAATAAATAAATATCCCC**G**	20			
*Alu*		F	GGTTAGGTATAGTGGTTTATATTTGTAATTTTAGTA	36	60	2/1	98
		R	ATTAACTAAACTAATCTTAAACTCCTAACCTCA	33			
		Pm	CCTACCTTAACCTCCC	16			

^1^ All tMDS probes were designed to contain the 6-Carboxyfluorescein fluorescent (6-FAM) in the 5′ end and the quencher ZEN-3′ Iowa Black^®^ FQ in the 3′ end of the oligo (5′-/56-FAM/ZEN/3IABkFQ/-3′). The *Alu* probe was designed to contain the fluorescent Cy5 in the 5′ end and the quencher TAO-3′ Iowa Black^®^ RQ-Sp in the 3′ end of the oligo (5′-/5-Cy5/TAO /3IAbkRQSp/-3′). ^2^ Guanines highlighted in bold bind to complementary methylated cytosines of interest. F: forward; R: reverse; Pm: probe specific for the methylated allele.

**Table A2:** Method performance per MethyLight assay, including minimum DNA input to detect methylation assessed by the methylated DNA standard serial dilution; average standard deviation (SD) of methylation detection; and linearity equations for normalizing qPCR methylation data assessed by DNA standards with known methylation levels.

Assay	Min DNA Input (ng)	Average SD	Equation for Data Normalization
**CpG1**	0.078	0.036	*y* = 0.8664*x* + 0.0182
**CpG2**	0.625	0.027	*y* = 1.0412*x* + 0.0308
**CpG3**	0.313	0.026	*y* = 1.056*x*
**CpG4**	0.078	0.010	*y* = 1.0325*x* + 0.0348
**CpG5**	0.0625	0.023	*y* = 0.9772*x* − 0.0302
**CpG6**	0.078	0.020	*y* = −0.5173*x*^2^ + 1.5932*x* − 0.0643
**CpG7**	0.625	0.020	*y* = 1.0135*x* + 0.0613
**CpG8**	0.078	0.022	*y* = 0.5787*x*^2^ + 0.4094*x* + 0.0314
**CpG8**	0.156	0.025	*y* = 0.9843*x* + 0.014
**CpG10**	0.078	0.034	*y* = 1.0439*x* − 0.0177
**CpG11**	0.078	0.025	*y* = 0.9695*x* − 0.0221
**CpG12**	0.156	0.036	*y* = −0.1247*x*^2^ + 1.1728*x* − 0.1337
**CpG13**	0.078	0.044	*y* = 1.1769*x* − 0.0989
**CpG14**	0.156	0.024	*y* = 0.8913*x* + 0.0585
**CpG15**	0.156	0.038	*y* = −1.2737*x*^2^ + 2.5978*x* − 0.3106
**CpG16**	0.078	0.027	*y* = 1.2423*x* − 0.0023
**CpG17**	0.078	0.026	*y* = 1.0117*x* + 0.029
**CpG18**	0.625	0.018	*y* = 0.9449*x* + 0.023
**CpG19**	0.078	0.036	*y* = 1.0686*x* − 0.0282
**CpG20**	0.078	0.030	*y* = 0.9236*x* + 0.0198
**CpG21**	0.156	0.031	*y* = 0.4054*x*^2^ + 0.6791*x* − 0.0246
**CpG22**	0.078	0.033	*y* = 0.9962*x* + 0.043
